# Histogram-based analysis of diffusion-weighted imaging for predicting aggressiveness in papillary thyroid carcinoma

**DOI:** 10.1186/s12880-022-00920-4

**Published:** 2022-11-02

**Authors:** Ran Wei, Yuzhong Zhuang, Lanyun Wang, Xilin Sun, Zedong Dai, Yaqiong Ge, Hao Wang, Bin Song

**Affiliations:** 1 Department of Radiology, Minhang Hospital, Fudan University, 170 Xinsong Road, Shanghai, 201199 People’s Republic of China; 2 GE Healthcare, Shanghai, People’s Republic of China

**Keywords:** Magnetic resonance imaging, Diffusion-weighted imaging, Apparent diffusion coefficient, Papillary thyroid carcinoma, Aggressiveness

## Abstract

**Background:**

To assess the potential of apparent diffusion coefficient (ADC) map in predicting aggressiveness of papillary thyroid carcinoma (PTC) based on whole-tumor histogram-based analysis.

**Methods:**

A total of 88 patients with PTC confirmed by pathology, who underwent neck magnetic resonance imaging, were enrolled in this retrospective study. Whole-lesion histogram features were extracted from ADC maps and compared between the aggressive and non-aggressive groups. Multivariable logistic regression analysis was performed for identifying independent predictive factors. Receiver operating characteristic curve analysis was used to evaluate the performances of significant factors, and an optimal predictive model for aggressiveness of PTC was developed.

**Results:**

The aggressive and non-aggressive groups comprised 67 (mean age, 44.03 ± 13.99 years) and 21 (mean age, 43.86 ± 12.16 years) patients, respectively. Five histogram features were included into the final predictive model. ADC_firstorder_TotalEnergy had the best performance (area under the curve [AUC] = 0.77). The final combined model showed an optimal performance, with AUC and accuracy of 0.88 and 0.75, respectively.

**Conclusions:**

Whole-lesion histogram analysis based on ADC maps could be utilized for evaluating aggressiveness in PTC.

## Background

Papillary thyroid carcinoma (PTC) represents the commonest pathological type of thyroid cancer, constituting 65–92.8% of all thyroid malignant tumors [1, 2]. PTC generally has a good prognosis [3], with 1–2% mortality and a survival rate above 99% for less-aggressive PTC [4]. Aggressive PTC requires a different clinical treatment strategy from non-aggressive PTC. The 2015 ATA guidelines [5, 6] recommend ipsilateral lobectomy instead of total thyroidectomy for low-risk PTC, and refraining from prophylactic central neck lymph node dissection, to avoid unnecessary complications. However, for aggressive PTC, total thyroidectomy and prophylactic central lymph node dissection are required, often with subsequent radioactive iodine-131 treatment. Currently, the aggressiveness of tumors can only be assessed by pathologically evaluating specimens obtained by thyroidectomy [7]. Therefore, preoperative evaluation of PTC aggressiveness is very important for determining the clinical treatment [6]. The determination of PTC invasiveness comprises of several different aspects, including the presence of thyroid capsule invasion, regional lymph node and distant metastases, and a special pathological subtype.

Ultrasound is the first method of choice for the examination of thyroid lesions [8, 9], but has certain limitations, including difficulty in assessing retrotracheal lymph nodes, a low specificity in the diagnosis of capsular invasion, especially minimal extrathyroidal extension (ETE) [10, 11], and a high dependence on the surgeon’s skills [6]. Fine-needle aspiration (FNA) biopsy is an essential method to obtain pathological specimens before surgery, but provides limited data on invasiveness due to very little tissue obtained, and cannot be used as a diagnostic criterion for invasiveness [12].

Diffusion-weighted imaging (DWI) is a widely applied functional imaging method, which uses the diffusion of water molecules to quantitatively analyze lesions without the use of contrast agents [13]. Apparent diffusion coefficient (ADC) is a quantitative index of DWI, which can reflect proliferation activity and cell count in different tumors. ADC has certain value in predicting the preoperative grade of tumors [13–15]. A previous study showed that ADC could accurately discriminate between malignant and benign thyroid tumors [16]. ADC is related to aggressiveness in PTC [17]. Furthermore, ADC is an effective tool for evaluating the aggressiveness and can predict extrathyroidal extension [18].

However, the majority of previous studies were subjective and lacked repeatability because ADC was calculated by a manually selected single region of interest (ROI). In addition, PTC is heterogeneous, and ADC largely depends on the delineated ROI, with possible incomplete assessment. ADC histogram assessment represents a more objective approach for examining ADC value distribution throughout the tumor, avoiding the subjectivity of ROI selection and ensuring reproducibility of measurements. Also, histogram analysis of ADC values can objectively reflect the overall molecular characteristics of a lesion. ADC histogram assessment can be used to evaluate invasiveness in prostate cancer [19], and to distinguish invasive from non-invasive meningiomas [20]. A previous report [21] indicated that ADC values and histogram analysis could predict different histopathological features in thyroid cancer. Histogram analysis of DWI was useful for prediction of lymphatic metastatic spread, proliferative activity, and cellularity in thyroid cancer.

Therefore, this study aimed to explore the predictive performance of histogram analysis of ADC maps in assessing PTC aggressiveness.

## Methods

### Patients

This retrospective study examined consecutive patients with thyroid nodules initially diagnosed by ultrasonography (US) between January 2019 and March 2021. Based on the American College of Radiology Thyroid Imaging, Reporting, and Data System [22], the tumor grades were TR3-TR5.

**Fig. 1:**
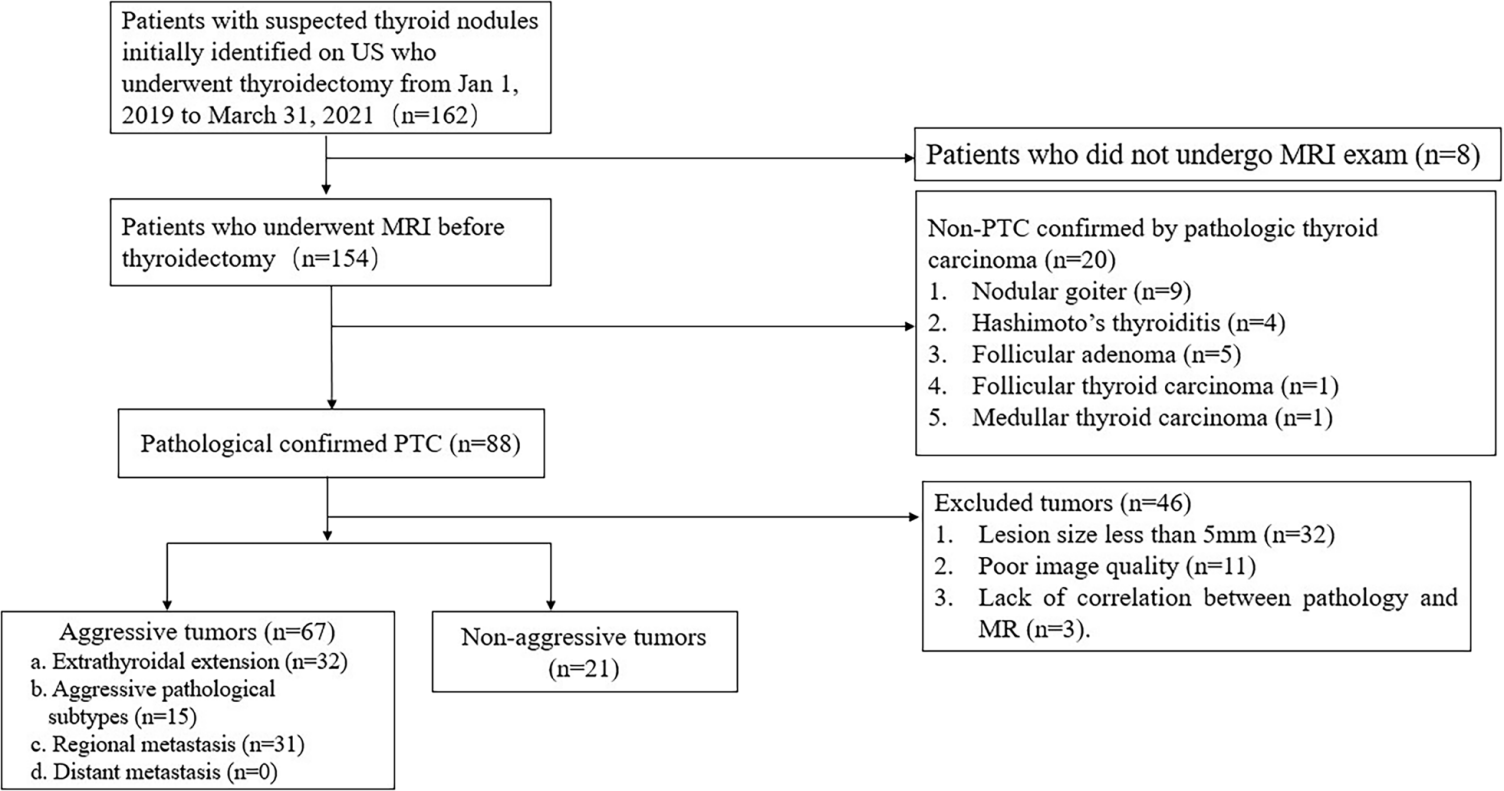
Study flowchart. *US* Ultrasound; *MRI* Magnetic resonance imaging; *PTC* Papillary thyroid carcinoma

Multi-parametric MRI was conducted on all the patients, with subsequent thyroid surgery (subtotal or total thyroidectomy) within seven days post-MRI. Pathological confirmation of PTC was obtained based on surgical specimens. Exclusion criteria were: (1) pathology not reflecting PTC; (2) tumor size < 5 mm; (3) completely different pathological and MRI data for tumor samples; (4) poor MR image quality. Finally, 88 patients were enrolled in this study. Figure 1 shows the study flowchart.

This study was approved by the Institutional Review Board of Minhang Hospital, Fudan University (approval number: 2020-008-01 K), and written informed consent was waived because of the retrospective study design.

### MRI acquisition

An EXCITE HD 1.5 T scanner (GE Healthcare, USA) with an 8-channel special neck surface coil was utilized to examine the patients as follows: (1) Axial T2-weighted (T2WI) fast recovery fast spin-echo with fat suppression: echo time (TE), 85 ms; repetition time (TR), 3000 ms; slice thickness, 4 mm; matrix, 320 × 224; spacing, 0.5 mm; field of view (FOV), 25 cm; number of excitations (NEX), 4. DWI with a single-shot echo planar imaging (EPI) sequence: minimal TE; TR, 6550 ms; slice thickness, 4 mm; matrix, 128 × 128; spacing, 0.5 mm; FOV, 25 cm; NEX, 6 (b value of 800 s/mm^2^). Spatial saturation bands were utilized to remove signals from overlying fat and adjacent tissues.

### Histopathological analysis

Surgical tumor specimens were assessed by a pathologist with > 10 years of related experience. Paraffin-embedded specimens were sectioned and stained with hematoxylin and eosin (H&E). Thereafter, the pathologist evaluated aggressiveness by histology based on set criteria. All individuals were then grouped into the aggressive and non-aggressive categories. Also, expression of Ki-67 and tumor-stromal ratio were assessed.

PTC aggressiveness was examined based on the American Thyroid Association (ATA) 2015 risk stratification system for differentiating thyroid carcinoma [6]. Expression of Ki-67 and tumor-stromal ratio were assessed by calculating the proportion of all the sections of the lesion.

### Image processing and analysis

Tumor segmentation ITK-SNAP (http://www.itk-snap.org) was utilized for thyroid tumor segmentation. Totally 88 regions of interest (ROIs) were manually delineated on ADC maps by two radiologists with 10 and 13 years of experience, respectively. Consensus was reached by discussion in case of discrepancy. ROIs were drawn slice-by-slice to reflect the tumor’s 3D volume. The largest tumors were assessed in various patients for reducing potential bias with many lesions in a given patient and improving the applicability of results.

For inter-observer agreement assessment, 30 random cases were chosen to calculate intraclass correlation coefficients (ICCs) for select parameters. Reliability was characterized as follows: (1) ICC < 0.4, poor; (2) ICC 0.41–0.60, medium; (3) ICC 0.61–0.80, good; (4) ICC > 0.80, excellent. Various features were utilized for further extraction, with ICCs reaching 0.80 [23].

Radiomic features were automatically extracted with the AK software version 3.2.2 (GE healthcare). First, the Mann–Whitney U test was used to examine whether the features had significant inter-group difference. Next, univariate logistic regression analysis was performed to assess whether the parameters could distinguish the two groups. The feature subset was selected with mRMR method, retaining features with minimum redundancy maximum relevance. Multivariate logistic regression analysis was conducted to build the prediction model. In the model development, tenfold cross-validation was used for evaluating the predictive performance. In the process of cross-validation, we used 90% data to train the model, and the remaining 10% data were used to evaluate the model performance. Meanwhile, the training and testing set were independent cohort, and all parameters of the model are determined by the training data. Then, we repeated above process. A total of 10 times cross-validation were implemented. Then, we repeated above process 10 times. The model’s performance in detecting aggressiveness of PTC was assessed by receiver operating characteristic (ROC) curve analysis, determining the area under the curve (AUC), sensitivity, specificity, accuracy, and negative and positive predictive values. Then, we presented the average performance of testing set.

## Results

### Patient features

Totally 88 patients aged 43.99 ± 13.51 years (range, 13–71 years) were included in the final analysis. According to pathology results, 67 (mean age, 44.03 ± 13.99 years) and 21 (mean age, 43.86 ± 12.16 years) cases were in the aggressive and non-aggressive groups, respectively. There were no statistically significant differences in Ki-67 expression and tumor-stromal ratio between the two groups. The characteristics of the included PTC cases are summarized in Table 1.

**Table 1:** Characteristics of patients in the aggressive and non-aggressive groups

	Aggressive group (n = 67)		Non-aggressive group (n = 21)		*p* value
Age(years)	44.03 ± 13.99		43.86 ± 12.16		0.96
Diameter(mm)	1.41 ± 0.71		0.93 ± 0.36		0.004
*Sex*					
Female	48		18		0.312
Male	19		3		
*Location*					
Right lobe	43		12		0.214
Isthmus of thyroid	4		0		
Left lobe	20		9		
Ki-67(%)	3.15 ± 8.41		2.38 ± 2.82		0.523
Tumor-stromal ratio (n)	low	high	low	high	0.418
	42	25	12	9	

### PTC aggressiveness prediction

**Fig. 2:**
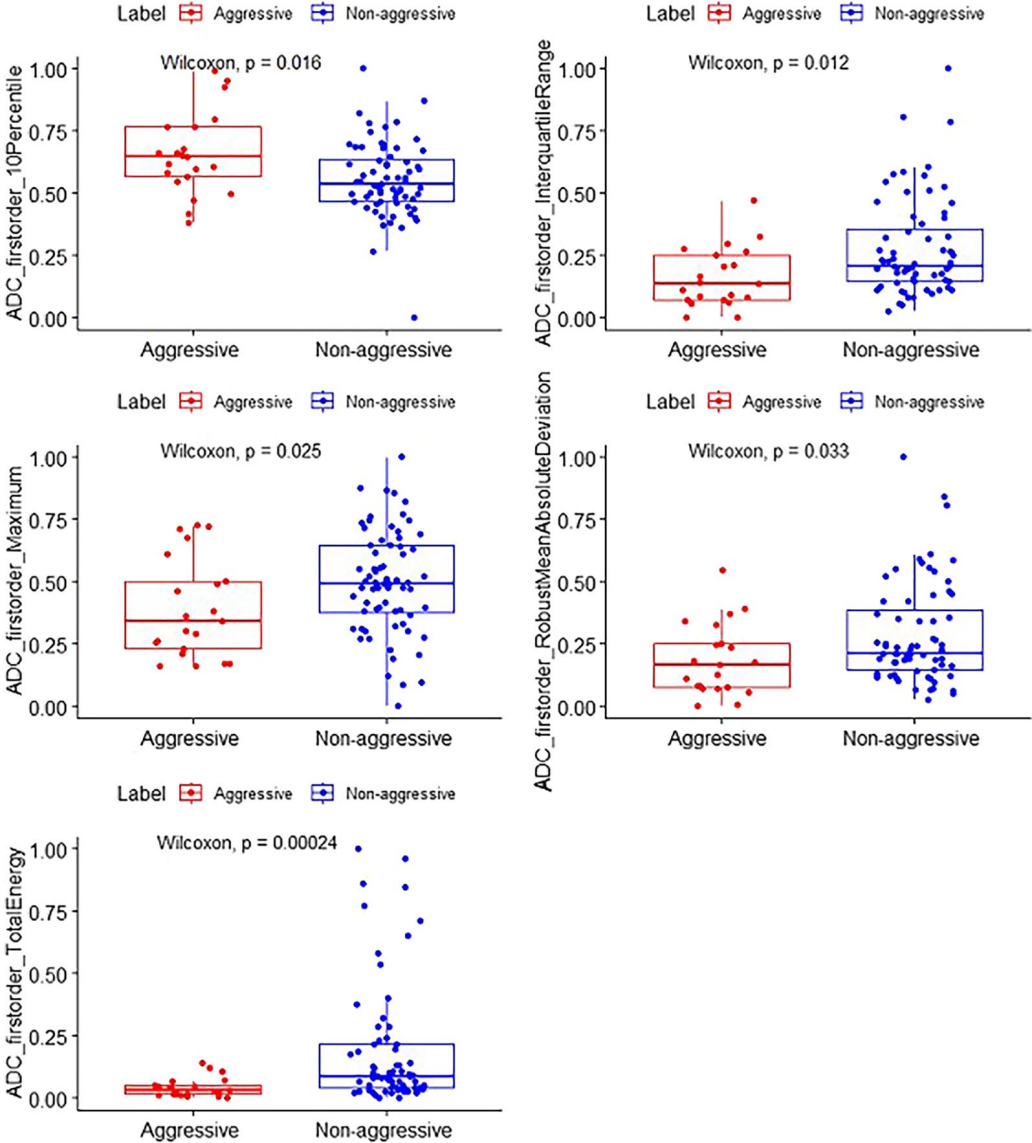
Scatterplots of ADC-derived histogram features. Red points represent aggressive PTCs, and blue points represent non-aggressive PTCs. Dotted lines show the best cutoffs of various histogram parameters for distinguishing aggressive and non-aggressive cases

**Fig. 3:**
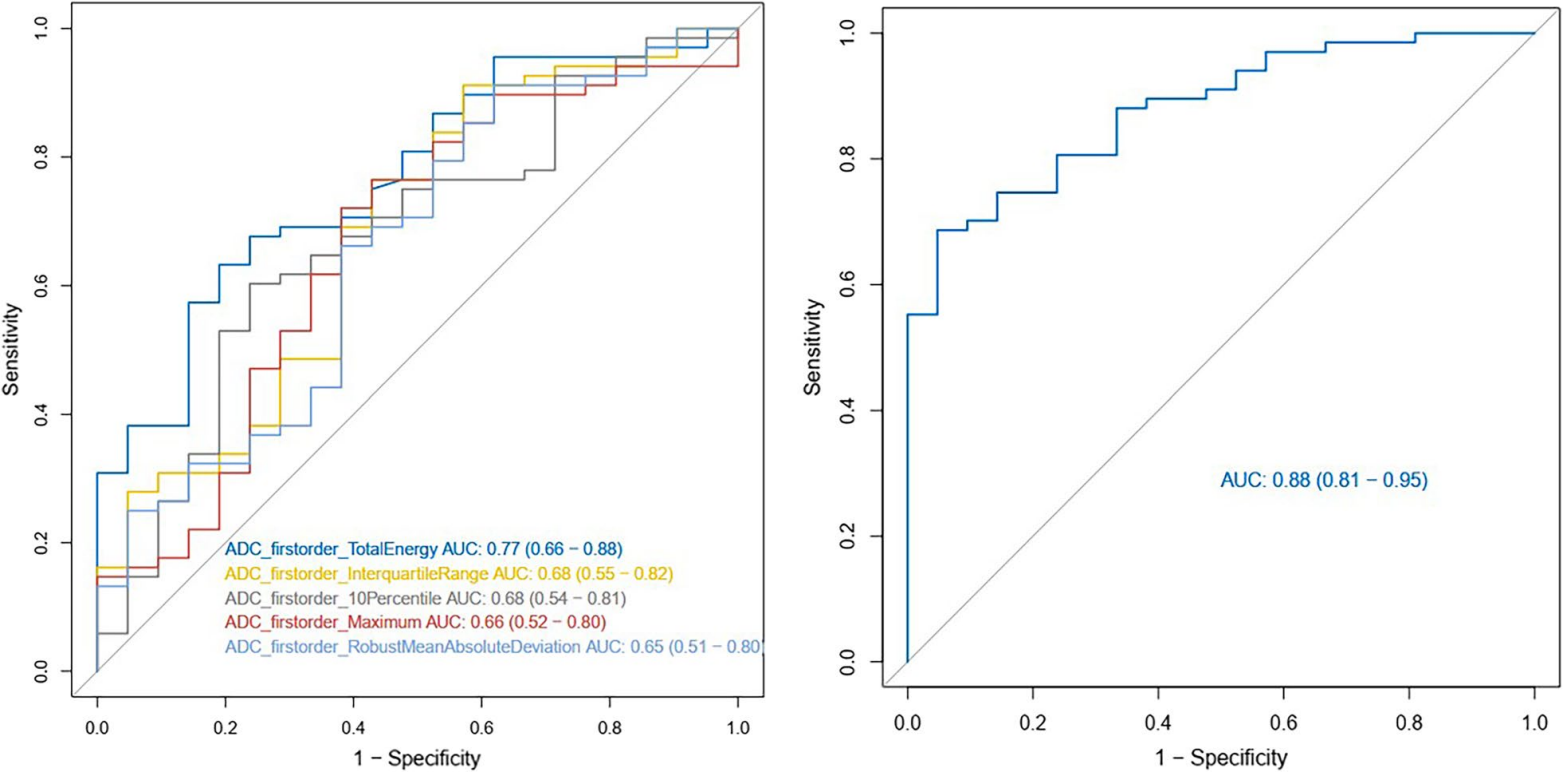
Predictive performances of significant histogram features and the final model

A total of 16 features were extracted. Ten features were significant by univariate logistic regression (*p* < 0.05), and five independent discriminative features were included in the final prediction model by multivariable logistic regression. Table 2 shows the odds ratios of the 10 features. Figure 2 depicts the scatterplots of select features in the two groups. Figure 3 depicts ROC curves for the five significant features, as well as the final model, in differentiating aggressive and non-aggressive lesions. The prediction model had an AUC of 0.88 (95% CI 0.81–0.95). Table 3 shows the model’s diagnostic performance: sensitivity, specificity and accuracy were 0.69, 0.95 and 0.75, respectively, and positive and negative predictive values were 0.98 and 0.49, respectively.

**Table 2:** Significant features for distinguishing aggressive and non-aggressive cases

Variable	Odds Ratio	Lower	Upper	*p*-value
ADC_firstorder_10Percentile	0.497888	0.288727	0.858571	0.012
ADC_firstorder_Energy	39,147,832	19.75277	7.76 × 10^13^	0.018
ADC_firstorder_InterquartileRange	2.691298	1.174322	6.167885	0.019
ADC_firstorder_Maximum	1.738082	1.015623	2.97446	0.044
ADC_firstorder_MeanAbsoluteDeviation	2.106290	1.07302	4.134552	0.030
ADC_firstorder_Minimum	0.258376	0.117799	0.566709	0.000732
ADC_firstorder_Range	4.222259	1.801889	9.893769	0.000916
ADC_firstorder_RobustMeanAbsoluteDeviation	2.040072	1.01126	4.115554	0.046
ADC_firstorder_TotalEnergy	39,147,832	19.75277	7.76 × 10^13^	0.018
ADC_firstorder_Variance	2.711680	1.096916	6.703531	0.031

**Table 3:** Predictive performances of significant variables and the final model

Variable	AUC(95% CI)	Accuracy	Sensitivity	Specificity	PPV	NPV
ADC_firstorder_TotalEnergy	0.77 (0.66–0.88)	0.67	0.63	0.81	0.91	0.40
ADC_firstorder_InterquartileRange	0.68 (0.55–0.82)	0.80	0.91	0.43	0.84	0.60
ADC_firstorder_10Percentile	0.68 (0.54–0.81)	0.64	0.60	0.76	0.89	0.37
ADC_firstorder_Maximum	0.66 (0.52–0.80)	0.70	0.72	0.62	0.86	0.41
ADC_firstorder_RobustMeanAbsoluteDeviation	0.65 (0.51–0.80)	0.79	0.91	0.38	0.83	0.57
Predictive model	0.88 (0.81–0.95)	0.75	0.69	0.95	0.98	0.49

## Discussion

This study demonstrates that histogram assessment of the ADC map, a non-invasive tool, could predict aggressiveness of PTC. Five features were selected for the final model. The results achieved moderate performance, with an accuracy of 0.75 and an AUC of 0.88 in predicting PTC aggressiveness. ADC_firstorder_TotalEnergy was the optimal histogram feature, with an AUC of 0.77.

Radiomics has been widely applied in predicting clinical prognosis, pathological grading and response to treatment since it permits quantitative assessment of intra-tumor parameters, transforming them into high-throughput parameters, mainly comprising histogram and texture features [24, 25]. Histogram analysis through conversion of MRI-based parameters in primary tumors could successfully detect aggressiveness in multiple lesions [26, 27]. DWI is an effective non-invasive imaging approach for evaluating tumor characterization, which represents the physiological characteristics. Previous studies [13, 16] indicated that ADC was useful in tumor grade prediction and detection. Hu et al. [18] showed ADC’s associations with ETE feature. Another study [28] reported that ADC values are associated with cervical lymph node metastasis. This study aimed to examine whole-lesion histogram analysis based on ADC maps to assess its ability to predict the aggressiveness of PTC. Subsequently, a predictive model was built with an improved performance in predicting tumor aggressiveness (AUC of 0.88). The above finding indicates histogram analysis of ADC maps may provide more biological data and constitute a better surrogate imaging-derived tool for detecting PTC aggressiveness. Additionally, histogram assessment may better meet the clinical needs, given its easy implementation and data interpretation without requirement of expert mathematical knowledge.

Routine DWI is unreliable for providing good image quality of thyroid because of susceptibility to motion artifacts, potentially rendering lesion determination difficult. Herein, we utilized the reduced FOV diffusion strategy instead of routine DWI to image the thyroid, which provides high-resolution and high-quality DWI for small structures [29–31]. An 8-channel special neck surface coil was used to allow higher image quality while reducing susceptibility to artifacts and distortions around the thyroid. In addition, ADC obtained according to manually selected ROIs is very subjective and variable. In this study, whole-lesion histogram assessment was utilized to examine the whole tumor, eliminating sample bias and enhancing the evaluation of intra-tumor heterogeneity [19, 32–34]. ADC_firstorder_TotalEnergy and ADC_firstorder_InterquartileRange showed strongly reduced values in aggressive PTC compared with non-aggressive cases. The discrepant ADC histogram features may reflect histopathological differences between aggressive and non-aggressive PTCs. For example, more severe desmoplastic response and higher cell density in aggressive PTCs reduce diffusion and lower the ADC values, while follicle and extracellular fluid abundance as well as reduced cell density in non-aggressive cases increase the ADC values. These findings indicated that greater the heterogeneity of tumor cellularity, the more aggressive the PTC, reflected by ETE, nodular metastasis and aggressive histopathology. A previous study [21] also showed that ADC and DWI kurtosis imaging correlate with extracellular changes, which was consistent with our results.

The results demonstrated no statistically significant differences in the Ki-67 expression and tumor-stromal ratio between the aggressive and non-aggressive groups. The reason was that PTC is an indolent tumor, and the expression of Ki-67 is relatively low [35]. PTC shows aggressive properties, including extrathyroidal extension (ETE), lymph node and distant metastases, or special pathological types. To the best of our knowledge, the tumor-stromal ratio could be associated with lymph node metastasis. Therefore, this study did not include Ki-67 and tumor-stromal ratio into the prediction model.

This study had several limitations. First, the sample size was small (88 cases), which could result in selection bias due to exclusion criteria of small tumor size and poor image quality. Advances in MRI might help to detect smaller PTC lesions and achieve high image quality. Second, another selection bias may exist because some PTC cases who underwent ultrasound examination without MRI were not enrolled in this study. Third, for predicting PTC aggressiveness, ADC values were not compared with other imaging features, including diffusion kurtosis imaging (DKI), which have also been utilized to assess thyroid nodules and related histological features. Nevertheless, these results were encouraging, and whole-lesion histogram analysis deserves popularization and wide application because it is convenient to use as a non-invasive imaging marker for predicting aggressiveness and therapeutic outcome in PTC.

## Conclusions

Overall, whole-lesion histogram analysis based on ADC maps is a non-invasive and quantitative tool, which may help to assess aggressiveness in PTC. Future larger-sample and independent multi-center studies are warranted to explore the potential clinical values of the histogram features detected in this study.

## Data Availability

The datasets analyzed in this study are available from the corresponding author on request.

## Abbreviations

ADC: Apparent diffusion coefficient
PTC: Papillary thyroid cancer
MRI: Magnetic resonance imaging
AUC: Area under the curve
ETE: Extra-thyroidal extension
DWI: Diffusion-weighted imaging
ROI: Regions of interest
T2WI: T2-weighted imaging
TE: Echo time
TR: Repetition time
FOV: Field of view
NEX: Number of excitations
EPI: Echo planar imaging
ICC: Intraclass correlation coefficient
CI: Confidence interval
ROC: Receiver operating characteristic
DKI: Diffusion kurtosis imaging
